# Sex‐specific genetic susceptibility to Alzheimer's disease and diabetes

**DOI:** 10.1002/alz70855_103096

**Published:** 2025-12-24

**Authors:** Alexander M. Kulminski, Fan Feng, Irina Kulminskaya, Alireza Nazarian, Elena Loiko, Yury Loika

**Affiliations:** ^1^ Social Science Research Institute, Duke University, Durham, NC, USA

## Abstract

**Background:**

Although Alzheimer's disease (AD) is more prevalent in women than men, women's survival advantage may not fully account for the AD‐related sex disparities. With studies suggesting a high genetic heritability for AD, exploring the genetic architecture behind sex differences in susceptibility to AD and its risk factors could enhance the understanding of disease mechanism.

**Method:**

We conducted genome‐wide association studies (GWAS) and pleiotropic meta‐analysis of AD and its risk factor—diabetes mellitus (DM)—in men and women of European ancestry. The AD GWAS focused on 172,097 men (including 8,445 cases) and 204,904 women (11,376 cases) from 10 studies. The DM GWAS included 165,287 men (24,154 cases) and 195,190 women (16,885 cases) from seven studies. The analyses leveraged strong genetic associations with DM to uncover weaker associations with AD.

**Result:**

GWAS of DM identified 30 men‐specific loci on 15 chromosomes characterized by the genome‐wide significant effects in men *p*_men_<5×10^‐8^ and weak effects with *p*_women_>10^‐4^ in women. Of them, there were eight loci mapped to *THADA, NYAP2, IGF2BP2, HLA, DGKB, NUCB2, HMGA2*, and *CLEC14A* gene clusters with men‐specific pleiotropic effects in which associations with AD attained *p*_men_<0.05 and *p*_women_>0.05. For women, we identified 16 loci on 11 chromosomes with *p*_men_>10^‐4^ and *p*_women_<5×10^‐8^. Women‐specific pleiotropic associations (*p*_men_>0.05 and *p*_women_<0.05) were observed in two gene clusters (*HNF1B, CDKN2B*). Female carriers of the *APOE* ε4 allele were at significantly higher AD risk (β=1.29, 95% confidence interval [CI]=1.24;1.33) than male carriers (β=1.12, CI=1.06;1.17). The ε4 allele was favorably associated with DM in both men (β=‐0.094, CI=‐0.121;‐0.066, *p* = 1.47×10^‐11^) and women (β=‐0.1065, CI=‐0.139;‐0.074, *p* = 1.53×10^‐10^), with no significant difference in the effects between sexes. The ε2 allele was favorably associated with AD in both men (β=‐0.756, CI=‐0.865;‐0.646, *p* = 1.41×10^‐41^) and women (β=‐0.805, CI=‐0.903;‐0.707, *p* = 2.09×10^‐58^). There was nether significant difference in the AD effects between sexes nor the ε2 allele was significantly associated with DM (*p*>0.3).

**Conclusion:**

Genetic susceptibility to both conditions, DM and AD, is more pronounced in men than women. Antagonistic associations the *APOE* ε4 allele with AD and DM and sex‐specific differences in the effects among AD and DM indicate a critical role of heterogeneity in the *APOE*‐related AD‐DM risk.

